# How to Properly Measure a Current-Voltage Relation?—Interpolation vs. Ramp Methods Applied to Studies of GABA_A_ Receptors

**DOI:** 10.3389/fncel.2016.00010

**Published:** 2016-02-02

**Authors:** Tushar D. Yelhekar, Michael Druzin, Urban Karlsson, Erii Blomqvist, Staffan Johansson

**Affiliations:** Section for Physiology, Department of Integrative Medical Biology, Umeå UniversityUmeå, Sweden

**Keywords:** current-voltage relation, voltage clamp, reversal potential, conductance, concentration changes, interpolation, voltage ramp, ion channel

## Abstract

The relation between current and voltage, I-V relation, is central to functional analysis of membrane ion channels. A commonly used method, since the introduction of the voltage-clamp technique, to establish the I-V relation depends on the interpolation of current amplitudes recorded at different steady voltages. By a theoretical computational approach as well as by experimental recordings from GABA_A_-receptor mediated currents in mammalian central neurons, we here show that this interpolation method may give reversal potentials and conductances that do not reflect the properties of the channels studied under conditions when ion flux may give rise to concentration changes. Therefore, changes in ion concentrations may remain undetected and conclusions on changes in conductance, such as during desensitization, may be mistaken. In contrast, an alternative experimental approach, using rapid voltage ramps, enable I-V relations that much better reflect the properties of the studied ion channels.

## Introduction

Ion channels are of fundamental importance to cellular function. They provide means of rapid signaling by effects on membrane voltage and on ion concentration in the vicinity of the membrane (for review, see e.g., Hille, 2001). The functional properties of ion channels are commonly studied under voltage-clamp conditions, which enable a systematic analysis of currents with respect to voltage (Hodgkin et al., 1952). The relation between current and voltage (I-V relation) may give information on conductance (see e.g., Hodgkin and Huxley, 1952a; Dodge and Frankenhaeuser, 1959; Bormann et al., 1987), gating properties (see e.g., Hodgkin and Huxley, 1952b; Frankenhaeuser, 1959; Bormann et al., 1987), ion selectivity (see e.g., Frankenhaeuser, 1962; Bormann et al., 1987; Chen et al., 1999) and/or ion concentrations (see e.g., Moran et al., 1980; Johansson et al., 1996; Karlsson et al., 2011) depending on the conditions and known parameters in a particular study.

Analysis of the I-V relations obtained from the current amplitudes at steady voltages, and often after different times after a voltage step or start of agonist application that activate particular channel types, has been frequently used since the introduction of the voltage clamp technique (Hodgkin et al., 1952) to understand the functional properties of various types of ion channels, including neuronal voltage-gated (for examples, see Frankenhaeuser, 1962; Johansson and Århem, 1992; Williams et al., 1997) and ligand-gated channels (Trussell et al., 1988; Mittman et al., 1990; Zhang and Trussell, 1994; Fu et al., 1997; Kaila et al., 1997; Karlsson et al., 1997a; Kumar and Huguenard, 2001; Bianchi and Macdonald, 2002; Christophe et al., 2002; Cordero-Erausquin et al., 2005), non-neuronal voltage-gated (Miyazaki et al., 1975; Yatani et al., 1987; Smith et al., 2002) and ligand-gated (Magleby and Stevens, 1972; Ifune and Steinbach, 1992; Sims, 1992) channels as well as other channel types (Zhang et al., 1998). As an alternative to recording the current at a steady voltage, I-V relations have also been obtained by using voltage ramps, where the controlled voltage changes gradually at a steady rate and simultaneously the current is continuously recorded (for examples concerning voltage-gated channels, see Fishman, 1970; Spindler et al., 1999; Del Negro et al., 2002; Yamada-Hanff and Bean, 2013; for ligand-gated channels see Bolton, 1975; Adams and Sakmann, 1978; Sims, 1992; Karlsson et al., 2011; for other channel types, see Zhang et al., 1998). The purpose of the present study is (i) to reveal critical differences between the I-V relations obtained using steady voltages and those obtained with voltage ramps and (ii) to clarify the significance of those differences for interpreting channel properties and ion concentrations, on basis of several examples of GABA_A_-receptor mediated membrane currents.

**Figure 1:**
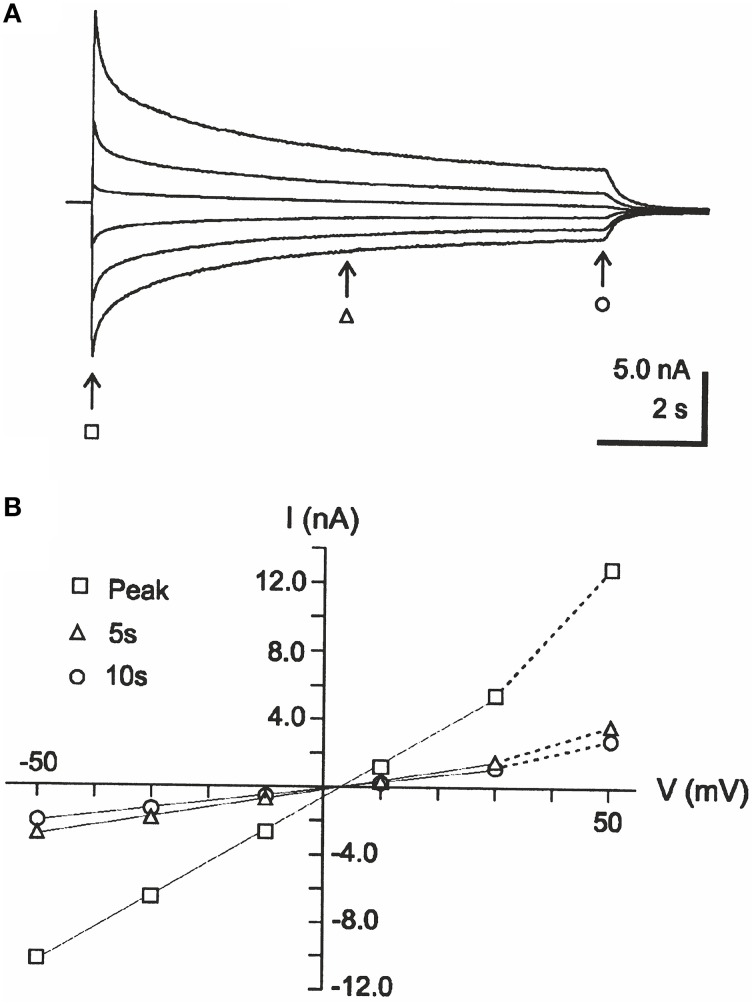
**Example of interpolation-type of I-V analysis, taken from Bianchi and Macdonald (2002)**. **(A)** Currents evoked by 10-s applications of 1 mM GABA to fibroblasts expressing GABA_A_ receptor subunits α1, β3, and γ2L, at steady voltages of 50, 30, 10, −10, −30, and −50 mV. **(B)** I-V relations corresponding to the currents in **(A)**, at the time points indicated by arrows and corresponding symbols. Note that the interpolated reversal potential does not change much with time. Reproduced from Bianchi and Macdonald (2002) with permission from John Wiley and Sons (Copyright of The Physiological Society 2002).

I-V relations obtained from currents at steady voltages have been used to clarify whether changes in ion concentrations take place during an experimental situation, for example, in studies of GABA_A_ receptor channels. Thus, with I-V relations obtained from the current amplitudes at different times after the start of agonist application and with application repeated at different steady voltages, the lack of substantial change in reversal potential with time has been taken to imply that ion concentration did not change significantly (Bianchi and Macdonald, 2002; see Figure 1, reversal potential ~3 mV for all I-V curves). The method used may be called the “interpolation method” because the estimate of reversal potential depends on the interpolation between current amplitudes measured at different voltages. In light of our recent study showing prominent changes in intracellular Cl^−^ concentration during GABA application (Karlsson et al., 2011), we were surprised to see that the interpolation method as used by Bianchi and Macdonald indicated that no concentration changes occurred during GABA application under roughly similar conditions, albeit in a different preparation. We therefore, hypothesized that the interpolation method fails to detect concentration changes that may be detected by other methods.

In the present study, we use a computational model to show that the interpolation method may give incorrect estimates of reversal potential and therefore conclusions on stable ion concentrations are not justified on basis of this method. The interpolation method may also provide a conductance (slope of the I-V relation) that does not correctly reflect the channel properties and may lead to mistaken conclusions on desensitization or inactivation. An alternative method, where reversal potential is obtained from the current during rapid voltage ramps (“voltage-ramp method”), in theory gives a much better estimate of the concentration of permeant ions as well as a conductance that reflects the channel properties.

We also confirm experimentally by perforated-patch recordings from rat preoptic neurons that the two methods give dramatically different I-V relations, where only those obtained with voltage ramps are correctly reflecting the conductance and the reversal potentials of currents through the studied channels. Some of the results have been presented in preliminary, abstract form (Yelhekar et al., 2014).

## Materials and methods

### Computational methods

Changes in intracellular Cl^−^ concentration and membrane currents during voltage-clamp conditions were computed numerically using the model developed by Karlsson et al. (2011), which was based on the description of synaptic GABA-mediated currents in medial preoptic neurons by Haage et al. (2005). In short, we used a linear three-state model with “unbound” (closed), “bound” (closed) and open states with a concentration-dependent rate constant for GABA binding (k_on_) of 3.0 [GABA] μM^−1^ s^−1^, for unbinding (k_off_) 723 s^−1^, for activation (transition from bound to open state; β) 2 500 s^−1^, and for deactivation (transition from open to bound state; α) 142 s^−1^, where [GABA] is GABA concentration.

$$\begin{array}{l} \text{Unbound}\overset{{\text{k}}_{\text{on}}}{\underset{{\text{k}}_{\text{off}}}{⇄}}\text{Bound}\overset{\beta }{\underset{\alpha }{⇄}}\text{Open} \end{array}$$

We, however, omitted the desensitized state in the description by Haage et al. (2005), to clarify the effect of internal Cl^−^ concentration ([Cl^−^]_i_) changes on the time course of current.

The fraction of channels in the unbound/closed (*C*), bound (*B*), and open (*O*) states, using the initial conditions *C* = 1, *B* = 0, and *O* = 0, were computed numerically as a function of time (*t*), by (forward) Euler integration using the equations:

(1) $$\begin{array}{rcl} \text{d}C∕\text{d}t & = & -{\text{k}}_{\text{on}}C+{\text{k}}_{\text{off}}\;B \end{array}$$

(2) $$\begin{array}{rcl} \text{d}B∕\text{d}t & = & {\text{k}}_{\text{on}}C-{\text{k}}_{\text{off}}\;B\;+\;\alpha \;O-\beta \;B \end{array}$$

(3) $$\begin{array}{rcl} O & = & 1-\left(C+B\right) \end{array}$$

The conductance (*G*), proportional to the fraction of open channels, current (*I*), and [Cl^−^]_i_ were computed according to the following equations:

(4) $$\begin{array}{r} G=G_{\text{max}}\;O \end{array}$$

(5) $$\begin{array}{r} I=G\left(V_{\text{m}}-E_{\text{Cl}}\right) \end{array}$$

Where,

$$\begin{array}{rcl} E_{\text{Cl}} & = & \left(\text{RT}∕\text{F}\right)\;\ln\;{\left[{\text{Cl}}^{-}\right]}_{\text{i}}∕{\left[Cl^{-}\right]}_{\text{o}}\;\left(\text{Nernst equation}\right),\\ stepequation \end{array}$$

(6) $$\begin{array}{rcl} \text{d}{\left[{\text{Cl}}^{-}\right]}_{\text{i}}∕\text{d}t & = & \tau_{\text{diff}}^{-1}\left({\left[{\text{Cl}}^{-}\right]}_{\text{pip}}-{\left[{\text{Cl}}^{-}\right]}_{\text{i}}\right)+I{\left({\text{Fv}}_{\text{equ}}\right)}^{-1}\left(1-T_{\text{Cl}}\right) \end{array}$$

where the first term represents the diffusional Cl^−^ exchange with the patch pipette according to Pusch and Neher (1988), the second term the exchange with pipette as a consequence of the electric current, and

$$\begin{array}{l} T_{\text{Cl}}={\left[{\text{Cl}}^{-}\right]}_{\text{i}}\;{\text{u}}_{\text{Cl}}{\left(\Sigma_{j}{\text{c}}_{j}|{\text{z}}_{j}|{\text{u}}_{j}\right)}^{-1}\;\left(transference\;number\;for\;Cl^{-}\right)\;\left(8a\right) \end{array}$$

with the ion concentrations (c), and corresponding charge numbers (z) for all ion species *j* used in the pipette-filling solution by Bianchi and Macdonald (2002) and corresponding ionic mobilities (u) taken from Ng and Barry (1995), Keramidas et al. (1999), Hille (2001) and the ionic mobility listing at http://web.med.unsw.edu.au/phbsoft/mobility_listings.htm, here giving

$$\begin{array}{l} T_{\text{Cl}}={\left[Cl^{-}\right]}_{\text{i}}\;7.92\;{\left({\left[Cl^{-}\right]}_{\text{i}}\;7.92+1244.62\right)}^{-1}\;\left(8b\right) \end{array}$$

v_equ_ = 2∕3 π r^3^ and denotes the “equivalent volume,” i.e., the cytosolic volume where Cl^−^ equilibrates, taken to be 50% of the total cell volume in a spherical cell of radius *r* (Karlsson et al., 2011), corresponding to the expected volume of the cytosol.

τ_diff_ = 78.4 R_pip_/DC_Cl_(r/7.68)^3^, the time constant for diffusional Cl^−^ exchange between cell and pipette, from Pusch and Neher (1988) with the pipette series resistance, R_pip_, assumed to be 10 MΩ and the diffusion constant for Cl^−^, DC_Cl_ = 203 10^−7^ cm^2^ s^−1^.

*V*_m_ is membrane potential, *E*_Cl_ equilibrium potential for Cl^−^, F the Faraday constant and T the temperature in *K* (= 295). Cl^−^ concentration on the outside ([Cl^−^]_o_) was assumed to be kept constant by the experimental perfusion system. Adaptation of the model to the conditions used by Bianchi and Macdonald (2002) was made by assuming [Cl^−^]_o_ = 164 mM, initial [Cl^−^]_i_ = 155 mM, cell radius = 7.5 μm and the maximum conductance, *G*_max_ = 280 nS, was chosen to achieve roughly the same peak current amplitude at +50 mV as observed experimentally by those authors.

Model adaptation to the experimentally studied preoptic neurons (see below) was made by adjusting [Cl^−^]_o_ to the experimental situation. Since the experiments were made with the amphotericin-B perforated patch technique where perforating pores have limited Cl^−^ permeability (~1/9 of the permeability to monovalent cations), the magnitude of the computational parameter R_pip_ (determining diffusional Cl^−^ exchange between cytosol and pipette) was increased nine times relative to the experimentally observed resistance. Initial [Cl^−^]_i_ was in these computations adapted to that measured by the voltage-ramp technique from a holding voltage close to the resting potential, and G_max_ was adapted to the measured conductance in those cells.

The integration time step was routinely 0.2 ms, but the stability of numerical computation was verified by similar responses with a time step of 0.02 ms. The computations were made using Turbo Basic software (Borland, Scotts Valley, CA, USA).

### Electrophysiological recording of membrane currents from preoptic neurons

**Figure 2:**
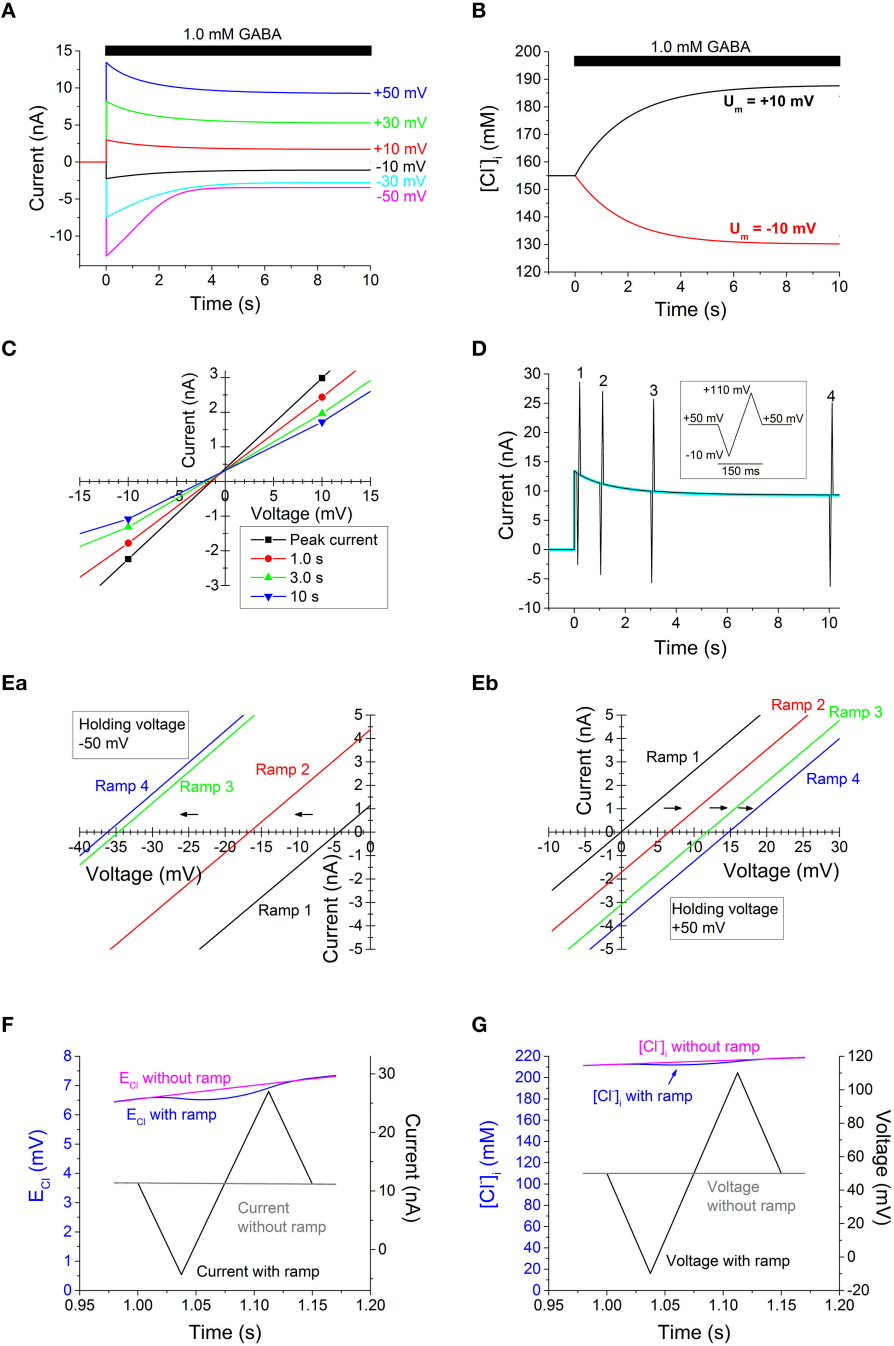
**Computed GABA-evoked currents, [Cl^−^]_i_ changes, and I-V relations on basis of data from Bianchi and Macdonald (2002) in the absence of any desensitization**. **(A)** Currents evoked by 1.0 mM GABA at the voltages indicated. Note the current decay. **(B)** Time course of [Cl^−^]_i_ change at the small voltages of +10 mV (top, black) and −10 mV (bottom, red). **(C)** Faulty I-V relations, obtained by interpolating currents at different voltages. Note that, erroneously, no or very small changes in the apparent E_Cl_ is suggested, in spite of the large changes in [Cl^−^]_i_ [shown in **(B)**]. **(D)** Current evoked by 1.0 mM GABA at +50 mV [as top current in **(A)**, which is here shown superimposed in cyan], but with voltage ramp sequences (inset; ±1.6 V s^−1^) at four different times. **(E)** I-V relations obtained from ramp sequences [as in **(D)**] from a steady voltage of −50 mV **(Ea)** and +50 mV **(Eb)**. Note that the dramatic shifts of *E*_*Cl*_ are detected with this method. Note also that the shifts occur in opposite direction for the “steady” voltages of −50 and +50 mV. For clarity, only part of the I-V curves around the last reversal potential are shown. **(F)** Time course of E_Cl_ and current at the time of ramp 2 in **(Eb)**, with superimposed plots for the cases with and without a voltage ramp. Note, that in the presence of a ramp, there is an initial drop in E_Cl_ during the first half of the ramp sequence, but that this is largely compensated by a rise in E_Cl_ during the later half, so that E_Cl_ returns to nearly the same level as without a ramp. **(G)** Time course of [Cl^−^]_i_ and voltage for the same ramp as in **(F)**. Note the small final effect of the ramp on [Cl^−^]_i_. Times given in (**A,B,D,F** and **G**) are relative to onset of GABA application.

**Figure 3:**
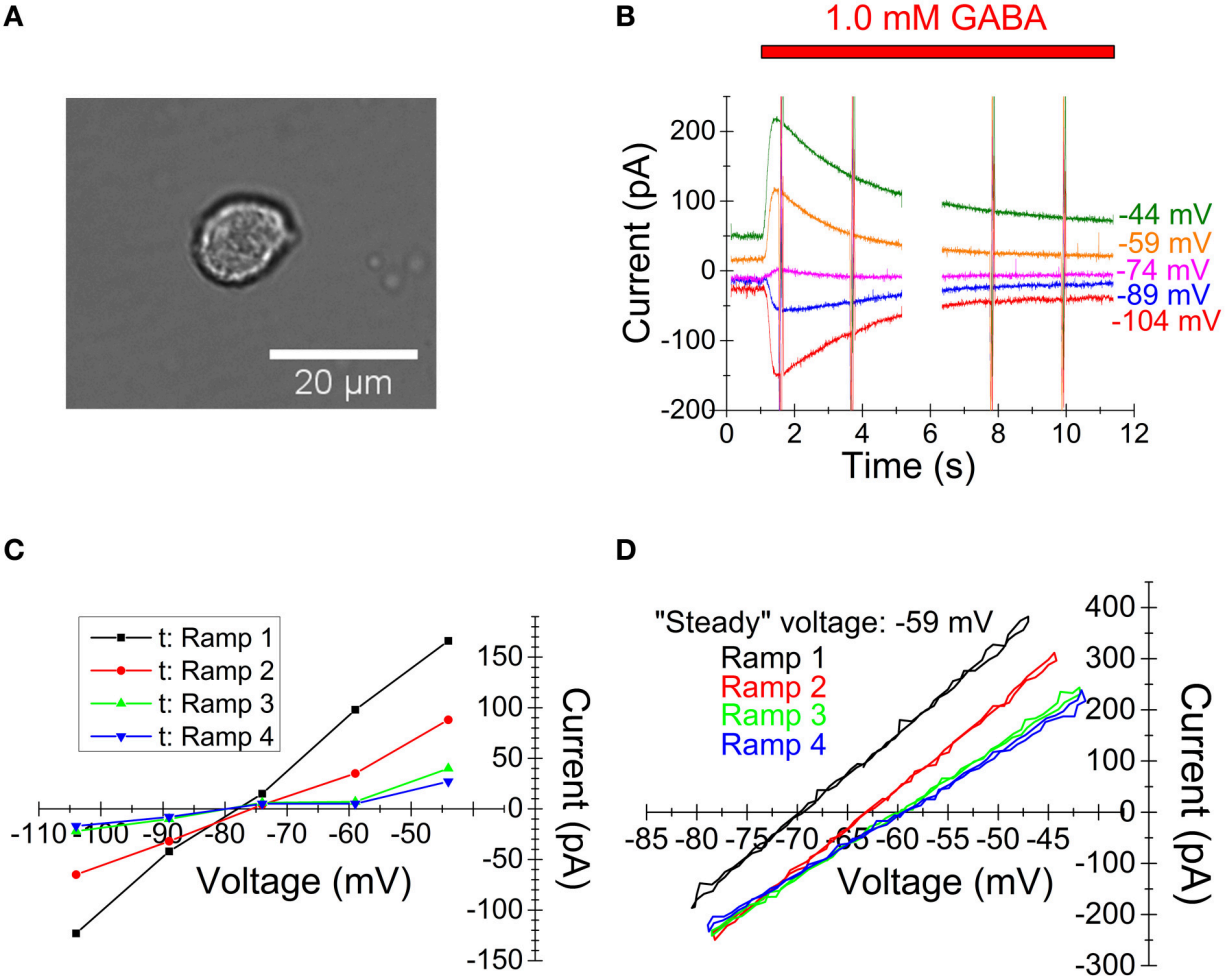
**Experimentally recorded membrane currents from preoptic neurons and corresponding I-V relations**. **(A)** Light-microscopic image of acutely dissociated preoptic neuron showing the typical shape of studied cells, selected for the absence of visible remaining neurites. **(B)** Raw currents in response to 1.0 mM GABA at the holding voltages indicated. Voltage ramp sequences of the type shown in Figure 2D (inset) were given at four times. (Current at ramps are truncated vertical lines due to plot scale. Data gap due to computer protocol limitations.) **(C)** I-V relations obtained by the interpolation method. Each curve corresponds to the time point just before a voltage ramp. Note the lack of clear change in reversal potential and reduced slope with time. **(D)** I-V relations obtained by the voltage-ramp method. Note the difference from **(C)** with clear changes in reversal potential and only small changes in slope. Note that currents from rising and decaying phases are superimposed and that there is a strict overlap, indicating that capacitive currents (which are of opposite polarity for rising and decaying phases) do not contribute significantly, in agreement with previous observations (Karlsson et al., 2011).

Acutely isolated neurons from the medial preoptic area of the brain of young (age 3–5 weeks) Sprague-Dawley rats were prepared by mechanical dissociation without enzymes (Karlsson et al., 1997a). Ethical permission was given by the regional ethics committee for animal research (“Umeå djurförsöksetiska nämnd,” approval no. A9-14). Solutions used for preparation were as described by Karlsson et al. (2011). In the present study, neurons without visible remaining neurites were chosen (**Figure 3A**), to avoid problems with poor voltage-clamp control (space clamp) of neurites. Whole-cell membrane currents were recorded with the amphotericin B-perforated patch method (Rae et al., 1991), as previously used to study membrane currents through GABA_A_ receptors in these neurons (Karlsson et al., 1997b). The extracellular solution contained (in mM): 137 NaCl, 5.0 KCl, 1.0 CaCl_2_, 1.2 MgCl_2_, 10 HEPES, and 10 glucose, pH 7.4 (NaOH). This solution was supplemented with 2.0 μM tetrodotoxin to block voltage-gated Na^+^ currents. For filling the recording (patch) pipettes, two alternative solutions, with low or high Cl^−^ concentration, were used. The “low [Cl^−^]” solution contained (in mM): 140 Cs-gluconate, 3.0 NaCl, 1.2 MgCl_2_, 10 HEPES, and 1.0 EGTA, pH 7.2 (CsOH). The “high [Cl^−^]” solution contained (in mM): 140 KCl, 3.0 NaCl, 1.2 MgCl_2_, 10 HEPES, and 1.0 EGTA, pH 7.2 (KOH). Amphotericin B (Sigma-Aldrich) was prepared from a stock solution (6 mg/100 μl DMSO) and added to a final concentration of 120 μg/ml of pipette-filling solution. Liquid junction potentials were calculated with the pClamp software (version 10, Molecular Devices, CA, USA) and compensated for in the voltages given. Recording equipment, including patch pipettes, was as described by Karlsson et al. (2011). Solutions were delivered through a custom-made perfusion system with alternative tubes merging into a common outlet and controlled by solenoid valves via a computer. The solution exchange time for 11 cells, not included in the study, but in identical conditions, has been measured to 53 ± 5 ms (mean ± SEM; Karlsson et al., 2011). After changing the steady (holding) voltage, a minimum of 30 s was allowed for stabilization of background currents before agonist application. For experiments with voltage ramps, a sequence of ramps at a rate of ±1.6 V s^−1^ (cf Figure 2D, inset) were applied. Ramp sequences were applied in the absence as well as presence of agonist, to enable subtraction of leak currents (cf Karlsson et al., 2011). All experiments were performed at room temperature (21–23°C).

The principles for the analysis and generation of I-V relations followed the description of Karlsson et al. (2011). In short, effects of series resistance (40 ± 4 MΩ) between the pipette and cytosol were calculated separately for currents generated in the absence and presence of agonist. The relation between leak current (and possible small voltage-gated current components) and voltage (after correction for series resistance) was established from the current recorded during a ramp in the absence of agonist and used to subtract leak currents from the current recorded during ramps in the presence of GABA. As previously noted (Figure S3 in Karlsson et al., 2011), leak and voltage-gated currents do not significantly contribute to estimated changes of reversal potential and [Cl^−^]_i_ (Voltage-gated K^+^ currents are reduced by the use of Cs^+^ in the “low [Cl^−^]” solution in the pipette and by the relatively small driving force for K^+^ in the experiments with “high [Cl^−^]” solution). In the conditions used, GABA-evoked current responses, conductances, and concentration changes measured in the studied cells are not altered by blocking GABA_B_ receptors (Karlsson et al., 2011).

With the HCO${}_{3}^{-}$-lacking, HEPES-buffered solutions used, the reversal potential for GABA-evoked currents, E_GABA_, is not significantly different from the Cl^−^ equilibrium potential, E_Cl_. Intracellular HCO${}_{3}^{-}$ concentration calculated by the Henderson-Hasselbalch equation [CO_2_ partial pressure from Boron (2009a), solubility constant and p*K* value from Boron (2009b) or Kajino et al. (1982)] was ~ 0.1 mM.

## Results

### Computed currents and I-V relations

To test the hypothesis that significant changes in Cl^−^ reversal potential should be expected during currents recorded under conditions as presented in Figure 1 (Bianchi and Macdonald, 2002), we first modified our previous simplified model (Karlsson et al., 2011) of GABA-evoked currents and adapted to the conditions used by Bianchi and Macdonald (2002) as described in the Materials and Methods. Importantly, the model included the concentration changes expected as a consequence of the Cl^−^ current. As previously, the model did not include any desensitization. For simplicity, we did not assume that any other channels or transporters contributed to changes in [Cl^−^]_i_. Note that we did not attempt to fit the detailed current kinetics, but aimed at answering the questions whether currents in roughly similar conditions may be expected to cause significant changes in [Cl^−^]_i_ and whether the interpolation and voltage ramp methods for constructing I-V curves and obtaining Cl^−^ reversal potentials would be appropriate for detecting such changes.

The computed current responses to 1.0 mM GABA, recorded at different steady voltages are shown in Figure 2A. As expected from our previous findings (Karlsson et al., 2011), the currents, after the rapid onset, showed a substantial decay in the continued presence of GABA, in spite of the absence of desensitization and in spite of the Cl^−^ exchange between cytoplasm and patch pipette. This decay was, even at the smaller voltages, accompanied by dramatic changes in [Cl^−^]_i_ (Figure 2B). In a biological experimental situation, the information on [Cl^−^]_i_ as presented in Figure 2B may not be available, but rather is inferred from the I-V relation. The I-V relation obtained by the interpolation method, with current amplitudes at the different voltages interpolated to a line, for each of the four time points after the start of GABA application, is shown at a magnified scale around zero current in Figure 2C. Note that the I-V curves corresponding to the different times show roughly similar “reversal” potential. If these reversal potentials are taken to reflect E_Cl_, the conclusion will be that [Cl^−^]_i_ did not change with time, contrary to the information presented in Figure 2B. In addition, it is clear that the I-V relations in Figure 2C show a conductance that decays with time. The conductance over the voltage interval shown in Figure 2C is 100% of the modeled conductance (261 nS) for the peak current, but 81% at 1.0 s, 63% at 3.0 s, and 54% at 10 s. If these conductance values are taken to reflect the channel properties, the conclusion will be that the channels desensitize, contrary to the modeled conductance that quickly (within a few ms) rises to a steady level of 261 nS (independent of voltage) without any subsequent decay.

The use of voltage ramps gives an alternative way to obtain information on the I-V relations. If ramp sequences are “symmetrical” with respect to the holding voltage (Figure 2D, inset), they allow for a current time course that, besides the ramp periods, does not differ much from the current in the absence of ramps (cf. superimposed currents with and without ramps in Figure 2D). I-V relations obtained from current responses to voltage ramps starting from a steady voltage of −50 and +50 mV are shown in Figures 2Ea,b (the latter corresponding to currents shown in Figure 2D), respectively. It is clearly seen that at the positive as well as negative starting voltage, the reversal potential changes dramatically with time, indicating very large changes in E_Cl_ and [Cl^−^]_i_. (When “symmetrical” ramps as shown in Figure 2D, inset, are used, two slightly different measurements of reversal potentials are obtained, corresponding to rising or decaying phases of the ramps. When ramps that do not cause substantial changes in [Cl^−^]_i_ are used, the difference is small or negligible. For clarity, Figure 2Eb shows I-V relations for the rising phase only, but the reversal potentials for rising and decaying phases differ by <0.1 mV for the first two ramps, ~0.1 mV for the third and ~0.5 mV for the fourth ramp.) The I-V curves obtained with the voltage-ramp method (Figures 2Ea,b) all deviate <1.3% from the correct modeled conductance, without artificial decay. (Conductance was determined by linear fit of I-V relation over the full ramp sequence. The small deviation that is present is likely a consequence of the (small) effect of the ramps on [Cl^−^]_i_.)

The currents associated with the voltage ramps used to probe the reversal potential and conductance will somewhat disturb E_Cl_ and [Cl^−^]_i_. However, with fast ramps, symmetrical around the holding voltage, the changes in E_Cl_ and [Cl^−^]_i_ are small (Figures 2F,G). The initial changes during the first half ramp sequence are largely compensated during the later half.

Thus, in theory, the I-V relations constructed with the common interpolation method fail to reflect the changes in reversal potential whereas I-V relations constructed on basis of the voltage ramp method do reflect such changes. In addition, the voltage ramp method does not give rise to the artificial reduction in conductance (slope of I-V relation) that is seen with the interpolation method. (The computations were based on a GABA-evoked conductance that did not desensitize.)

### Experimentally recorded currents and I-V relations

To verify that the theoretically obtained results above apply to living cells, we recorded membrane currents from acutely dissociated neurons (Figure 3A) from the medial preoptic area of rat brain. Current responses to 1.0 mM GABA were recorded at different holding voltages, with voltage ramp sequences applied at some intervals after the start of GABA application [see Materials and Methods; “low [Cl^−^]” solution used in pipette]. In similarity with the computed currents above and as expected from previous studies (Karlsson et al., 1997b, 2011), the recorded currents showed a substantial decay in the presence of GABA (Figure 3B). I-V relations generated by the interpolation method (at the times just before each ramp sequence) show small, if any, changes in reversal potential and a conductance that decays markedly with time (Figure 3C). The resolution for the reversal potential at later times (after GABA application) is poor due to the small current amplitudes.

I-V relations, from the same current records (Figure 3B), obtained by the voltage ramp method are clearly different (Figure 3D). In agreement with the theoretical results above, the reversal potential changes clearly with time, but the conductance changes only slightly with time (i. e. there is only little true desensitization). Also, the resolution for the reversal potential at later times is good. The same principle applied when a low [Cl^−^] (5.4 mM; *n* = 6) concentration and when a high [Cl^−^] (145.4 mM; *n* = 5) concentration was used in the pipette. (Note that, as described in the figure legend, capacitive currents do not contribute significantly to the I-V relations shown in Figure 3D.)

**Figure 4:**
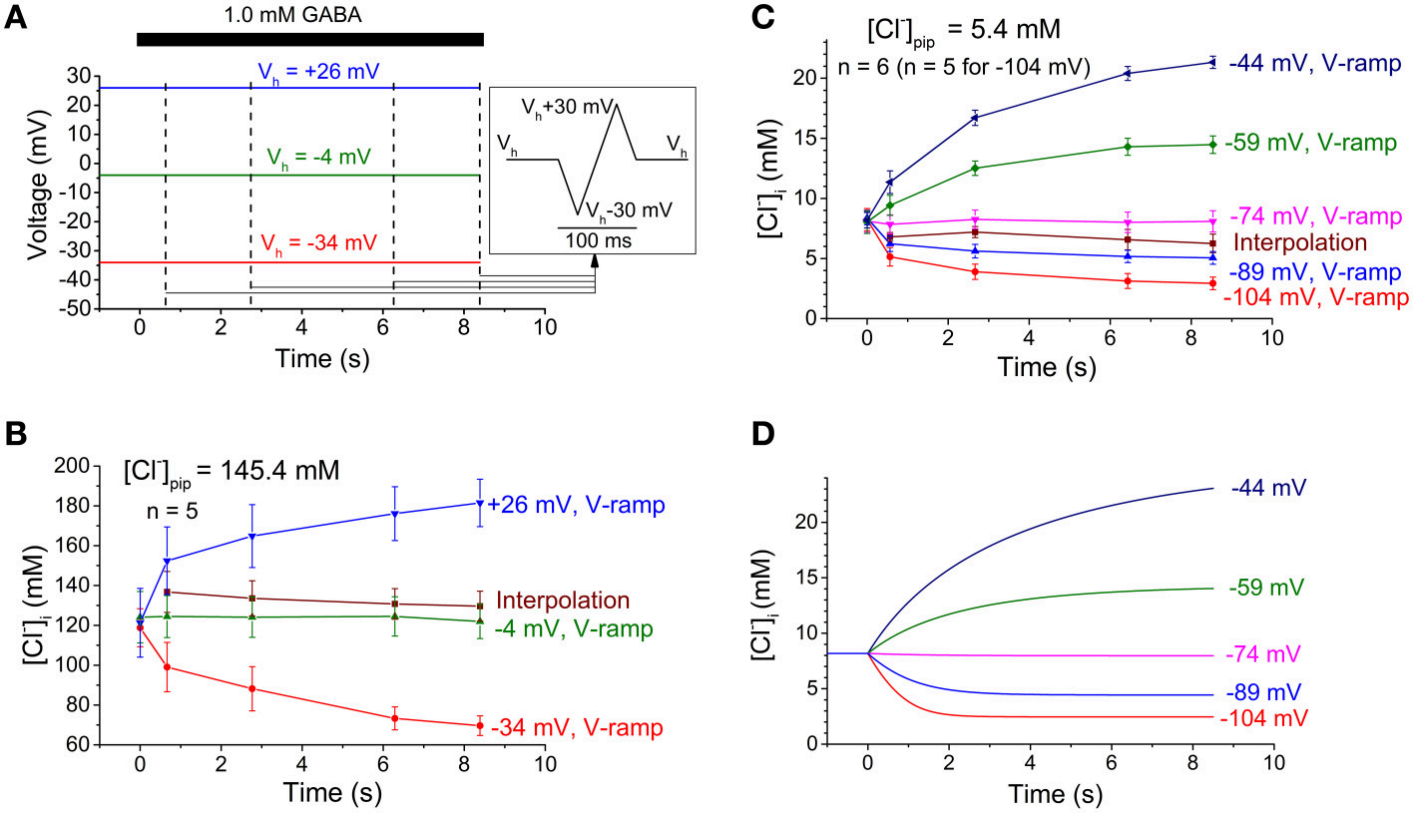
**Experimental protocol and changes in [Cl^−^]_i_**. (A) Experimental protocol used to estimate [Cl^−^]_i_ as presented in **(B)**. Dashed vertical lines indicate times of current measurement for interpolation and also the start of a voltage ramp sequence (inset, right). Note that the ramp sequence was added four times to each of the holding voltages (V_h_) used. GABA application indicated by the bar at the top. **(B,C)** Experimental [Cl^−^]_i_ estimates obtained with high **(B)** or low **(C)** Cl^−^ concentration in the pipette. [Cl^−^]_i_ obtained with the voltage ramp method (V-ramp) are given for each of the different holding potentials. [Cl^−^]_i_ obtained with the interpolation method depends on a construct from the data at different holding potentials and thus only one curve is obtained. Note the dramatic changes in [Cl^−^]_i_ obtained with the voltage ramp method, however, not detected with the interpolation method. Initial [Cl^−^]_i_ was estimated with the voltage-ramp method at the start of each experiment. Data points and error bars represent mean ± S.E.M. from 5 to 6 neurons, as indicated. **(D)** Time course of [Cl^−^]_i_ as obtained by the computational model when adapted to the preoptic neurons and the experimental conditions used for **(C)**. Note that the computational model predicts [Cl^−^]_i_ changes of a magnitude similar to that obtained from the experimental data analyzed with the voltage-ramp method.

Figure 4 summarizes the experimental paradigm (Figure 4A) and the changes in cytosolic Cl^−^ concentration ([Cl^−^]_i_) calculated from the I-V relations obtained with the two pipette solutions (Figures 4B,C). The [Cl^−^]_i_ calculated from reversal potentials obtained by the interpolation method did not change much with time whereas the [Cl^−^]_i_ calculated from reversal potentials obtained by the voltage-ramp method changed dramatically with time when the test voltage differed much from the initial E_Cl_. When the computational model was adapted (adjusted [Cl^−^]_o_, initial [Cl^−^]_i_, G_max_ and diffusional exchange between pipette and cytosol; see Materials and Methods) to fit the preoptic neurons from which the experimental data was obtained, it clearly showed that [Cl^−^]_i_ changes of a similar magnitude as estimated with the voltage-ramp method should be expected (Figure 4D).

## Discussion

The present study shows, by theoretical description as well as by biological experiments, that the two most common methods for plotting I-V relations, here called the interpolation and the voltage-ramp methods, may give dramatically different results. The interpolation method, which has been used since the introduction of the voltage-clamp technique (Hodgkin et al., 1952), gives rise to I-V relations that may not reflect the reversal potential of currents through the studied channels. In addition, the conductance obtained from the slope of the I-V relation may not reflect the channel properties and may for instance artificially show a decline in conductance with time, e.g., a desensitization, in the absence of true channel gating. The voltage-ramp method, on the other hand, does show changes in reversal potential and does not introduce artificial changes in conductance.

**Figure 5:**
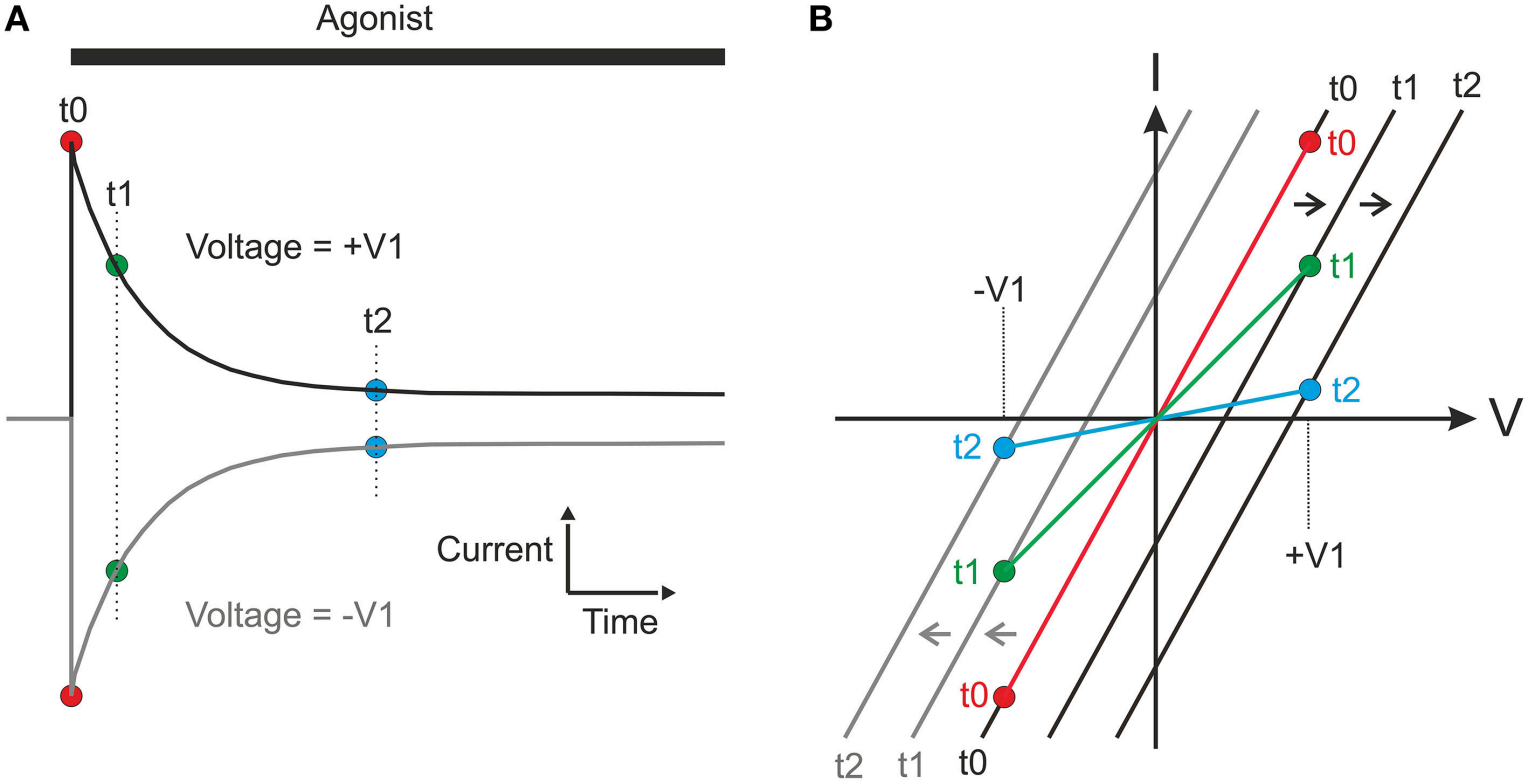
**Hypothetical experiment showing the principles underlying the incorrect conductances and reversal potentials obtained from the interpolation method**. For simplicity, symmetrical starting concentrations, and symmetrical volumes for equilibration on the two sides of membrane are assumed. **(A)** Agonist-induced currents obtained at two different holding membrane potentials [corresponding to +V1 and −V1 in **(B)**]. Filled circles represent current at time points (t0, t1, and t2) used for constructing I-V curve [in **(B)**] with interpolation method. **(B)** Black and gray lines represent true I-V relations at times t0–t2 at the positive (black) and negative (gray) voltage, as would have been detected by the voltage-ramp method. Filled circles represent current at t0–t2 as measured at the steady holding membrane potentials in **(A)** and the colored lines the corresponding I-V curves obtained by the interpolation method. Note that these curves fail to show the changes in reversal potential and that they indicate a slope (conductance) that artificially decreases with time.

Why do these differences occur and why does the interpolation method fail to detect changes in reversal potential and give an artificial conductance? Consider a hypothetical experiment, where a ligand gated channel is activated repeatedly at two different levels of membrane voltage (+V1 and −V1; Figure 5A). For simplicity, we assume starting conditions with symmetrical concentrations of permeant ions and instantaneous activation at time t0, but no subsequent desensitization. If the ion concentrations will change as a consequence of the ion flux, then the current will relax as shown in Figure 5A. In Figure 5B, the black and gray lines show the I-V relations expected from the-voltage-ramp method, correctly showing the changes in reversal potential and the constant conductance. If we assume that the peak current is reached before significant concentration changes occur, using the peak values (red filled circles at t0 in Figure 5) with the interpolation method will also result in a similar, correct conductance estimate. However, at later times (such as t1 or t2 in Figure 5), for each of the current traces, only a single voltage value (+V1 and −V1, respectively for the two traces) is available for use by the interpolation method. If a line is interpolated between the points for, e.g., t1 (filled green circles in Figure 5B) in the I-V plot, it is clear from the figure that this line will artificially indicate a reversal potential at zero and a reduced slope, indicating a reduced conductance. It may be noted that the principle illustrated does not depend on the magnitude of the voltages ±V1 and therefore, including more voltages will not change the reversal potential.

In a real experiment, channel activation is not instantaneous and some change in ion concentrations may already have occurred at the time of peak current. (In other cases, the current may be rising slowly throughout the studied time interval, without any well-defined peak.) In addition, the conditions for changes in ion concentrations are not symmetrical with respect to inside and outside. Further, some true desensitization often takes place. To clarify the true properties of channels studied and/or the changes in ion concentrations that occur as a result of ion flux under such conditions, it is critical to use I-V relations that are not misleading. This is the case if, for instance, channel desensitization is studied, since quantification of channel conductance (from the I-V relation) a significant time after the onset of activation is needed. As should be clear from above, I-V relations obtained with the voltage-ramp method are to be preferred over those obtained with the interpolation method whenever ion concentration changes may occur. In many cases where such changes are negligible, e.g., due to small ion flux with respect to cytosolic volume, the interpolation method may be used without problems.

There are, however, disadvantages/difficulties also with the voltage-ramp method. First, a ramp rate and voltage interval must be selected as to minimize unwanted changes in gating of voltage-sensitive channels (or indirect voltage-sensitivity of other channel types such as, e.g., NMDA receptors; see also below). Alternatively, in studies of ligand-gated channels, voltage-gated channels may be blocked. Otherwise, the gating of such channels may contribute to the conductance as well as the reversal potential obtained, in a way depending on the voltage-gated channel type present. Second, a ramp rate that is too low may be accompanied by unwanted changes in ion concentration during the voltage ramps, although symmetrical ramps with respect to holding voltage (or even around the expected reversal potential of the ionic current) as above may be used to minimize such changes. The idea is to shape the voltage ramp as to keep the total ionic flux (i.e., the time integral of ionic current) low, but sufficient for detection of conductance and reversal potential, during the ramp. Computer simulations as above may help in understanding under what conditions changes in concentrations should be expected.

In the cases described here, capacitive currents did not contribute significantly to the experimentally recorded currents (Figure 3; cf. Karlsson et al., 2011). It is possible, however, that capacitive currents would contribute more in cells with larger capacitive relative to ionic currents. Then, I-V curves for positive and negative ramp phases would be shifted in opposite direction due to the added capacitive current component (C_m_ d*V*_m_/d*t*, where C_m_ is membrane capacitance). Elimination of this component, which is constant for each ramp phase (disregarding the first sub-millisecond period influenced by the time constant R_pip_C_m_, if in the usual range of electrophysiological recording), is simple, e.g., by averaging the currents at positive and negative phases.

The description above concerned I-V relations based on currents through GABA_A_ receptors. Are similar principles to be expected also for other channel types? The main reason for the difference in I-V relations obtained with the two described methods was the effect of ion concentration changes during the relatively large and long-lasting currents (i.e., large ion flux with respect to cytosolic volume). Since, large and long-lasting currents of any ion species may cause concentration changes independent of the stimulus activating the channels, we cannot exclude that the described principles apply also to other channel types. Significant changes in intracellular ion concentration have been shown already at the peak of GABA- and glycine-activated currents (Karlsson et al., 2011), implying that the problems are not necessarily avoided by measuring peak currents. In many cases, however, the interpolation method likely works well when ion flux is limited, for example by rapid inactivation or desensitization, such as seen in many voltage-gated Na^+^ channels and AMPA receptors, respectively. On the other hand, voltage-gated K^+^ currents in several preparations may contribute significant currents for many seconds or even a minute after an activating voltage step (see e.g., Clay, 1989; Druzin et al., 2011). Further, large K^+^ currents may cause ion concentration changes not only in the cytosol, but also in the extracellular space between the neuronal membrane and glial cells (Frankenhaeuser and Hodgkin, 1956; Moran et al., 1980; Clay, 1989). It seems possible that I-V relations obtained by the interpolation method in studies of voltage-gated channels may be misleading in the situations when significant changes in ion concentrations occur. Such problems may be overcome by the voltage-ramp technique if ramps of a rate and amplitude that do not significantly alter channel gating are applied. (For example, as shown by Fishman (1970), a high ramp rate may be used to avoid activation of voltage-gated K^+^ currents.) In cases where this is difficult (e.g., due to fast gating kinetics), one should be aware of the potential problems with the interpolation method. If slow voltage ramps are used to study gating of voltage-gated channels, one should consider the possible changes in ion concentrations. In such situations, the I-V relation will reflect (i) changes in ion concentration as well as (ii) channel gating and (iii) conductance through open channels and may thus be difficult to interpret. Again, computer simulations of the type used above may help in understanding under what conditions changes in concentrations should be expected.

In conclusion, I-V relations obtained by the common interpolation method are associated with several problems: (i) reversal potentials may not reflect the current through the studied channels, (ii) resolution of measured reversal potentials may be poor, and (iii) apparent changes in conductance may not reflect the properties of the studied channels. I-V relations obtained with the voltage-ramp method, if properly designed, do better reflect the properties of the studied channels.

## Author contributions

TY contributed to study design, experimental recording, analysis, and paper writing, MD and UK contributed to study design, analysis, and paper writing, EB contributed to experimental recording and analysis, SJ contributed to idea, study design, computations, analysis, and paper writing.

### Conflict of interest statement

The authors declare that the research was conducted in the absence of any commercial or financial relationships that could be construed as a potential conflict of interest.
